# Evaluation of disease activity in patients with rheumatoid arthritis treated with tofacitinib by RAPID3: post hoc analyses from two phase 3 trials

**DOI:** 10.1007/s10067-018-4077-3

**Published:** 2018-04-14

**Authors:** Vibeke Strand, Eun Bong Lee, Yusuf Yazici, Ara Dikranian, Bethanie Wilkinson, Liza Takiya, Chuanbo Zang, Eustratios Bananis, Martin J. Bergman

**Affiliations:** 1 Division of Immunology and Rheumatology, Stanford University School of Medicine, Palo Alto, CA USA; 2 Seoul National University, Seoul, Republic of Korea; 3 Hospital of Joint Diseases, New York University, New York, NY USA; 4 Cabrillo Center for Rheumatic Disease, San Diego, CA USA; 5 Pfizer Inc, Groton, CT USA; 6 Pfizer Inc, Collegeville, PA USA; 7 Drexel University College of Medicine, Philadelphia, PA USA

**Keywords:** Low disease activity, RAPID3, Remission, Rheumatoid arthritis, Tofacitinib

## Abstract

Tofacitinib is an oral Janus kinase inhibitor for the treatment of rheumatoid arthritis. We evaluated the relationship between disease activity, according to Routine Assessment of Patient Index Data 3 (RAPID3) after 6-month treatment with tofacitinib, and long-term outcomes at 24 months. This was a post hoc analysis of two 24-month, phase 3, randomized controlled trials in methotrexate (MTX)-naïve (ORAL Start [NCT01039688]) or MTX-inadequate responder patients (ORAL Scan [NCT00847613]) receiving tofacitinib 5 or 10 mg twice daily (BID) as monotherapy or with background MTX. RAPID3 scores were calculated at baseline, month (M)6, and M24, and defined as remission (≤ 3), low (LDA; > 3–≤ 6), moderate (MDA; > 6–≤ 12), or high disease activity (HDA; > 12). Clinical Disease Activity Index (CDAI), Health Assessment Questionnaire-Disability Index (HAQ-DI) scores, and radiographic non-progression (modified Total Sharp Scores ≤ 0) at M24 were evaluated by M6 RAPID3 response. Among patients receiving tofacitinib 5 or 10 mg BID, respectively, 42.2 and 51.5% (ORAL Start) and 29.8 and 39.0% (ORAL Scan) achieved RAPID3 remission/LDA at M6. Most patients maintained/improved RAPID3 responses at M24. A higher proportion of patients in RAPID3 remission/LDA versus MDA/HDA at M6 achieved CDAI remission, reported normative HAQ-DI scores (< 0.5), and achieved both normative HAQ-DI scores and radiographic non-progression at M24. Patients achieving RAPID3 remission/LDA after 6-month treatment with tofacitinib 5 or 10 mg BID have improved long-term outcomes versus patients with MDA/HDA. These findings support the use of RAPID3 to monitor longer-term disease activity in conjunction with physician-assessed measures.

## Introduction

Rheumatoid arthritis (RA) is a chronic and debilitating autoimmune disease that affects approximately 0.4–1.3% of the global population [1]. RA is characterized by systemic inflammation, persistent synovitis, pain, and joint destruction. A treat-to-target approach for RA has been recommended by the American College of Rheumatology (ACR) and the European League Against Rheumatism (EULAR), with sustained remission, or at least low disease activity (LDA) if remission cannot be achieved, as the therapeutic target for treatment [2, 3]. There are six measures of RA disease activity currently endorsed by ACR: Routine Assessment of Patient Index Data 3 (RAPID3), Simplified Disease Activity Index (SDAI), Clinical Disease Activity Index (CDAI), Disease Activity Score 28, and Patient Activity Scale (PAS) or PASII [2].

RAPID3 is a patient-reported evaluation of disease activity based on a pooled index of the three ACR RA core dataset patient-reported outcomes (PROs): Patient Global Assessment of disease activity (PtGA), Patient Assessment of Arthritis Pain (PAIN), and Health Assessment Questionnaire-Disability Index (HAQ-DI) [4]. RAPID3 is a quick and easy means to assess disease activity, and patients can complete the assessment prior to meeting with a physician. RAPID3 requires less than one eighth of the time required to complete a 28-Joint Count Assessment [5], and its ease and rapidity in completion make it valuable for use in the clinical setting. Based exclusively upon PROs, it is frequently used to complement physician-assessed measures, rather than as a substitute for joint counts, or face-to-face visits with physicians [6].

Tofacitinib is an oral Janus kinase inhibitor for the treatment of RA. The efficacy and safety of tofacitinib 5 and 10 mg twice daily (BID) administered as monotherapy or in combination with conventional synthetic disease-modifying antirheumatic drugs, mainly methotrexate (MTX), in patients with moderately to severely active RA, have been demonstrated in phase 2 [7–11] and phase 3 [12–17] randomized controlled trials (RCTs) of up to 24-month duration and in long-term extension (LTE) studies with as long as 114 months of observation [18–21]. Improvements in PROs have been reported in phase 2 [22], phase 3 [23–27], and LTE [18, 28] tofacitinib studies.

Recently, the longer-term benefits of tofacitinib treatment, according to disease activity defined by CDAI at 6 months, were examined in two phase 3 RCTs: ORAL Start and ORAL Scan [29]. In this analysis, CDAI remission or LDA at month 6 was associated with attainment of normative HAQ-DI scores (< 0.5) and higher rates of radiographic non-progression by modified Total Sharp Scores (mTSS) (change from baseline ≤ 0) at month 24. The objective of this post hoc analysis was to evaluate disease activity at month 24 according to RAPID3 response at month 6, in patients with RA who received tofacitinib 5 or 10 mg BID as monotherapy or with background MTX in ORAL Start and ORAL Scan. We also examined disease activity (CDAI) and physical functioning (HAQ-DI), including the proportions of patients reporting normal physical functioning with no radiographic progression (mTSS) at month 24, based on RAPID3 responses at month 6.

## Patients and methods

### Study design

Data were analyzed from two phase 3, multicenter RCTs: ORAL Start (NCT01039688) [15] and ORAL Scan (NCT00847613) [16]. ORAL Start was a 24-month trial of tofacitinib monotherapy in MTX-naïve patients. Patients were randomized 2:2:1 to receive tofacitinib 5 mg BID, tofacitinib 10 mg BID, or MTX (at a starting dose of 10 mg per week, with 5 mg increments per week every 4 weeks, to 20 mg per week by week 8). ORAL Scan was a 24-month trial of tofacitinib in combination with MTX, in patients with an inadequate response to MTX (MTX-IR) [16]. Patients were randomized 4:4:1:1 to receive tofacitinib 5 mg BID, tofacitinib 10 mg BID, or placebo advanced to tofacitinib 5 or 10 mg BID, in combination with MTX. Patients receiving placebo who did not respond at month 3 (< 20% improvement in swollen and tender joint counts from baseline) were advanced blindly to tofacitinib 5 or 10 mg BID; at month 6, all remaining placebo patients were advanced to tofacitinib.

Both RCTs were conducted in accordance with the Declaration of Helsinki, International Conference on Harmonisation Guidelines for Good Clinical Practice and local country regulations. The trial protocols were approved by the Institutional Review Boards and/or Independent Ethics Committee at each trial center. All patients provided written, informed consent.

### Patients

Detailed inclusion and exclusion criteria for each trial have been previously reported. Patients were eligible if they were ≥ 18 years old with a diagnosis of active moderate to severe RA, according to ACR 1987 revised criteria [30].

### Assessments and outcomes

RAPID3 scores were calculated at baseline, months 6 (primary time point), and 24 (end of each trial). To calculate RAPID3 scores, each individual measure (PtGA, PAIN, and HAQ-DI) was scored from 0 to 10 for a total of 30. Disease activity levels for RAPID3 were defined as remission (≤ 3), LDA (> 3 to ≤ 6; excluding remission), moderate disease activity (MDA; > 6 to ≤ 12), and high disease activity (HDA; > 12).

CDAI responses and HAQ-DI scores were assessed at month 24, stratified by month 6 RAPID3 responses. Mean CDAI changes from baseline and the proportion of patients achieving categorical disease activity responses were analyzed. Disease activity levels according to CDAI were defined as remission (CDAI ≤ 2.8), LDA (excluding remission; CDAI > 2.8 to ≤ 10), MDA (CDAI > 10 to ≤ 22), and HDA (CDAI > 22). Normative physical function was defined as HAQ-DI score < 0.5. The proportion of patients reporting normative HAQ-DI scores with radiographic non-progression (defined as a change from baseline in mTSS ≤ 0) were evaluated.

### Statistical analysis

This analysis was post hoc and no adjustments were made for multiple comparisons. Non-responder imputation was used for the comparisons of RAPID3 remission and LDA (including remission) at months 6 and 24 between treatment and control groups. Observed cases were used for all other endpoints. Categorical endpoints were summarized by frequency (*n*, %). Continuous endpoints were descriptively summarized by mean and standard deviation. Efficacy endpoints were presented with 95% confidence intervals (CIs); the result was described as a numerical difference if the 95% CI overlapped. Significance level was set at *p* < 0.05 for comparisons of RAPID3 scores between tofacitinib and MTX or placebo. The full analysis set for CDAI, HAQ-DI, and mTSS assessments included patients with non-missing RAPID3 data at month 6.

## Results

### Patients

In total, 1729 patients were included in ORAL Start and ORAL Scan, of whom 1389 (80.3%) received tofacitinib. Patient demographics and baseline characteristics were generally similar between patient populations in each trial (Table 1), with the exception that patients in ORAL Start had shorter disease duration and had not received MTX in a therapeutic trial or in practice.

**Table 1:** Patient demographics and baseline characteristics in ORAL Start and ORAL Scan

	ORAL Start (*N* = 948)			ORAL Scan (*N* = 781)		
	Tofacitinib 5 mg BID (*N* = 370)	Tofacitinib 10 mg BID (*N* = 394)	MTX (*N* = 184)	Tofacitinib 5 mg BID + MTX (*N* = 316)	Tofacitinib 10 mg BID + MTX (*N* = 309)	Placebo + MTX (*N* = 156)
Female, *n* (%)	283 (76.5)	324 (82.2)	143 (77.7)	264 (83.5)	265 (85.8)	134 (85.9)
Age, years, mean (SD)	50.4 (12.2)	49.3 (12.8)	48.8 (13.3)	53.6 (11.6)	51.9 (11.5)	52.6 (11.7)
Disease duration, years, mean (range)	3.0 (0.0–44.0)	3.4 (0.0–34.0)	2.7 (0.0–30.0)	8.8 (0.25–43.0)	8.9 (0.25–42.0)	9.2 (0.42–43.5)
RAPID3						
Mean score (SD)	17.1 (6.0)	17.2 (5.8)	16.8 (5.9)	16.3 (5.9)	16.1 (5.9)	15.3 (6.1)
Remission (≤ 3), *n* (%)	4 (1.1)	4 (1.0)	0 (0.0)	3 (1.0)	8 (2.6)	3 (1.9)
LDA (> 3 to ≤ 6; no remission), *n* (%)	17 (4.6)	12 (3.1)	7 (3.8)	12 (3.8)	9 (2.9)	14 (9.0)
MDA (> 6 to ≤ 12), *n* (%)	44 (11.9)	62 (15.8)	29 (15.8)	63 (20.0)	55 (17.9)	25 (16.0)
HDA (> 12), *n* (%)	305 (82.4)	315 (80.2)	148 (80.4)	237 (75.2)	236 (76.6)	114 (73.1)
PAIN (VAS), mean (SD)	59.2 (23.9)	61.4 (23.1)	59.0 (23.6)	58.4 (14.0)	57.6 (14.5)	54.9 (13.1)
HAQ-DI, mean (SD)	1.5 (0.6)	1.5 (0.7)	1.5 (0.6)	1.4 (0.7)	1.4 (0.7)	1.3 (0.7)
PtGA, mean (SD)	60.4 (24.5)	60.9 (22.5)	57.9 (24.3)	58.0 (23.6)	56.4 (23.0)	54.1 (22.9)
CDAI, mean (SD)	39.3 (12.7)	38.2 (12.5)	39.1 (13.2)	35.5 (11.6)	34.9 (12.1)	34.3 (12.5)

*BID* twice daily, *CDAI* Clinical Disease Activity Index, *HAQ-DI* Health Assessment Questionnaire-Disability Index, *HDA* high disease activity, *LDA* low disease activity, *MDA* moderate disease activity, *MTX* methotrexate, *PAIN* Patient Assessment of Arthritis Pain, *PtGA* Patient Global Assessment of Arthritis, *RAPID3* Routine Assessment of Patient Index Data 3, *SD* standard deviation, *VAS* visual analogue scale

At baseline, 0.0–2.6% of patients were in RAPID3 remission, 2.9–9.0% LDA (excluding remission), 11.9–20.0% MDA, and 73.1–82.4% HDA across RCTs (Table 1).

### RAPID3 rates of remission and LDA

**Fig. 1:**
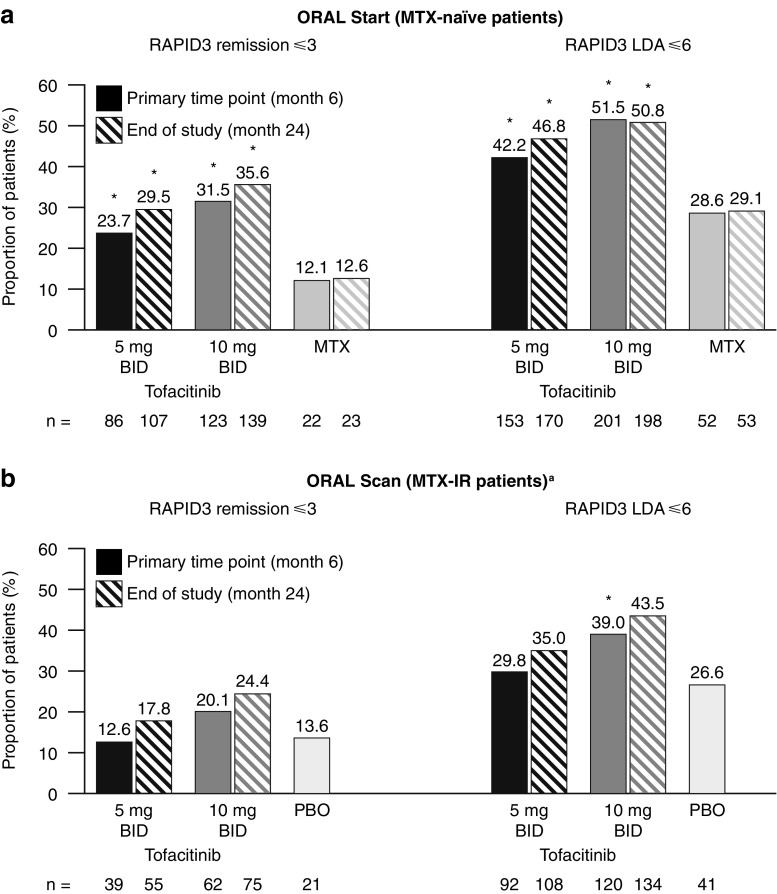
Rates of RAPID3 remission and LDA (including remission) at months 6 and 24 in **a** ORAL Start and **b** ORAL Scan. *p < 0.05 for tofacitinib versus MTX (ORAL Start—months 6 and 24) or placebo (ORAL Scan—at month 6). ^a^Patients received tofacitinib in combination with MTX. Non-responder imputation was used for missing values. Placebo data are not shown at month 24 for ORAL Scan, as those patients initially receiving placebo advanced to tofacitinib at month 3 or month 6. Statistical analysis for month 24 data was therefore not plausible. *BID* twice daily, *IR* inadequate response, *LDA* low disease activity, *MTX* methotrexate, *PBO* placebo, *RAPID3* Routine Assessment of Patient Index Data 3

In ORAL Start, significantly higher rates of RAPID3 remission and LDA were observed with tofacitinib 5 or 10 mg BID compared with MTX at months 6 and 24 (*p* < 0.05; Fig. 1a). In ORAL Scan, significantly higher rates of RAPID3 LDA were observed with tofacitinib 10 mg BID compared with placebo at month 6 (*p* < 0.05; Fig. 1b). LDA rates with tofacitinib 5 mg BID and remission rates for tofacitinib 10 mg BID were numerically higher than placebo in ORAL Scan, but not significantly different at month 6. In both RCTs, numerically higher rates of RAPID3 remission and LDA were observed with tofacitinib 10 mg BID than with 5 mg BID at months 6 and 24 (Fig. 1a, b). Higher rates of RAPID3 remission and LDA were observed at month 24 compared with month 6 for all treatment groups except for LDA rates with tofacitinib 10 mg BID (Fig. 1a, b).

### Longer-term outcomes based on RAPID3 responses at month 6

#### RAPID3 responses

**Fig. 2:**
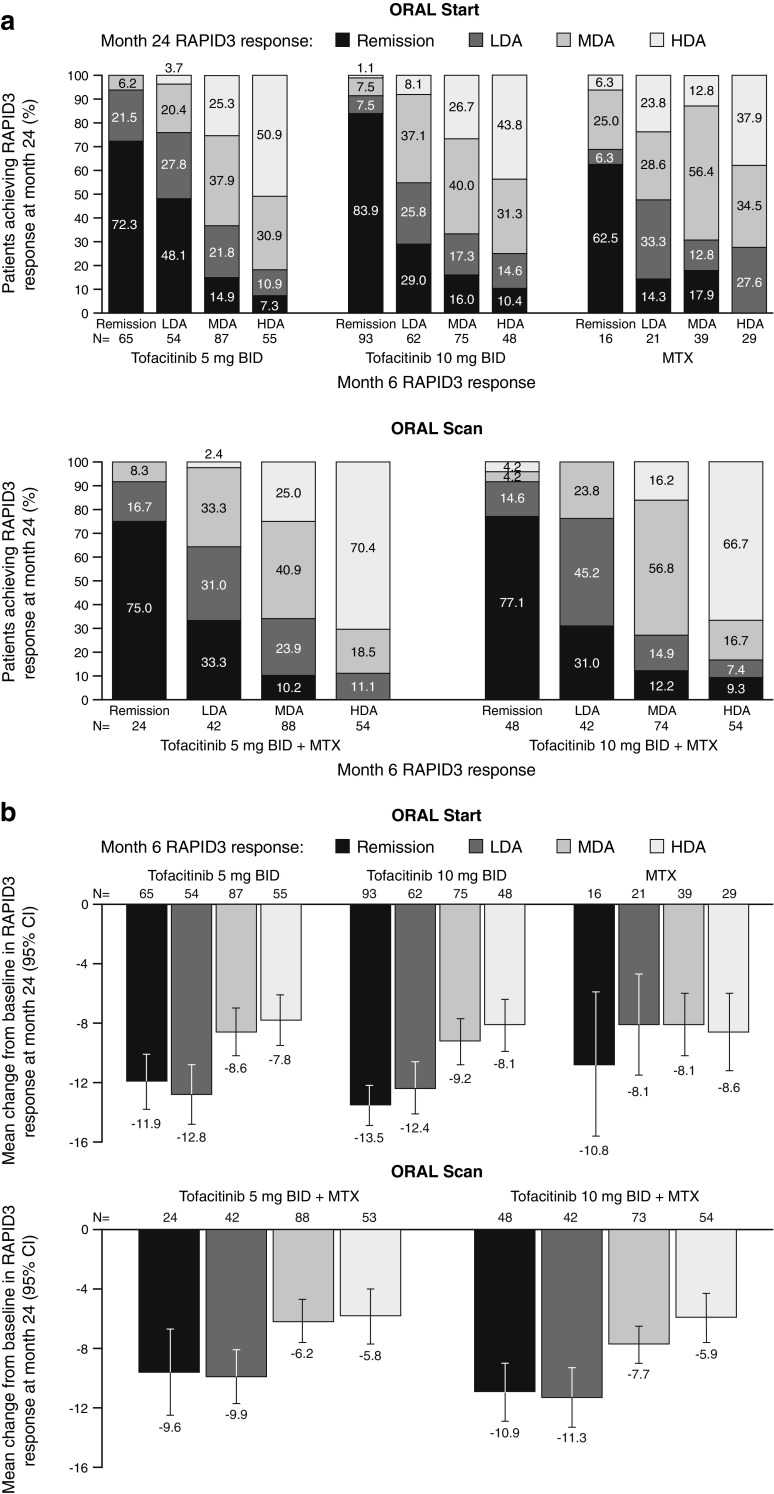
**a** Month 24 RAPID3 response stratified by RAPID3 responses at month 6. **b** Mean changes from baseline in RAPID3 at month 24 stratified by RAPID3 responses at month 6. Disease activity levels for RAPID3 score were defined as remission (≤ 3), LDA (> 3 to ≤ 6; excluding remission), MDA (> 6 to ≤ 12), and HDA (> 12). *BID* twice daily, *CI* confidence interval, *HDA* high disease activity, *LDA* low disease activity, *MDA* moderate disease activity, *MTX* methotrexate, *RAPID3* Routine Assessment of Patient Index Data 3

The majority of patients maintained or improved their RAPID3 response from month 6 to month 24 (Fig. 2a) in both RCTs. In ORAL Start, a higher proportion of patients receiving either dose of tofacitinib maintained remission and LDA at month 24 compared with patients receiving MTX.

Mean changes from baseline in RAPID3 scores at month 24 were greater in patients who achieved RAPID3 remission or LDA at month 6 in both RCTs compared with those who had achieved MDA or HDA at month 6 (Fig. 2b). Patients receiving tofacitinib who achieved RAPID3 remission or LDA at month 6 had numerically greater improvements in RAPID3 scores from baseline at month 24 than patients receiving MTX in ORAL Start (Fig. 2b).

#### CDAI response

**Fig. 3:**
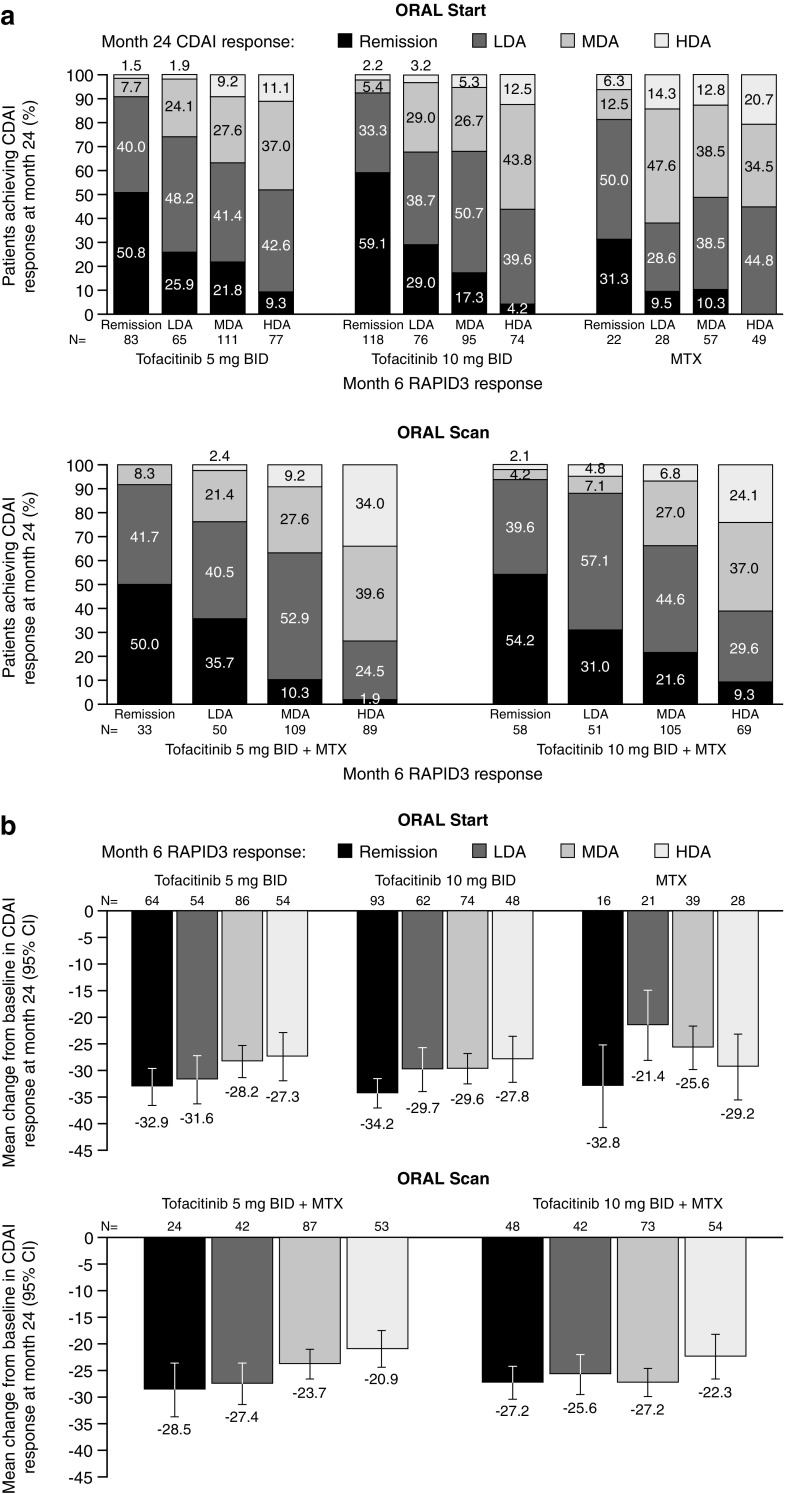
**a** Proportion of patients achieving CDAI responses at month 24 stratified by RAPID3 responses at month 6. **b** Mean changes from baseline in CDAI scores at month 24 stratified by RAPID3 responses at month 6. Disease activity levels for RAPID3 score were defined as remission (≤ 3), LDA (> 3 to ≤ 6; excluding remission), MDA (> 6 to ≤ 12), and HDA (> 12). Disease activity levels according to CDAI were defined as remission (CDAI ≤ 2.8), LDA (excluding remission; CDAI > 2.8 to ≤ 10), MDA (CDAI > 10 to ≤ 22), and HDA (CDAI > 22). *BID* twice daily, *CDAI* Clinical Disease Activity Index, *CI* confidence interval, *HDA* high disease activity, *LDA* low disease activity, *MDA* moderate disease activity, *MTX* methotrexate, *RAPID3* Routine Assessment of Patient Index Data 3

For both doses of tofacitinib, ≥ 50% of patients in RAPID3 remission at month 6 also achieved CDAI remission at month 24 in both RCTs (Fig. 3a). In contrast, approximately 31% of patients receiving MTX in ORAL Start in RAPID3 remission at month 6 also achieved CDAI remission at month 24. For those patients in RAPID3 LDA at month 6 of ORAL Start, the majority of patients achieved either CDAI remission or LDA at month 24 with tofacitinib 5 mg BID (26 and 48%, respectively) and 10 mg BID (29 and 39%, respectively), compared with a minority of MTX patients (10 and 29%, respectively).

Improvements in CDAI at month 24 were numerically greatest in patients in RAPID3 remission in both RCTs (Fig. 3b). In ORAL Start, patients receiving tofacitinib, who were in RAPID3 remission at month 6, had similar improvements from baseline in CDAI scores to those receiving MTX, while those with LDA at month 6 had numerically greater improvements (Fig. 3b).

#### HAQ-DI

**Fig. 4:**
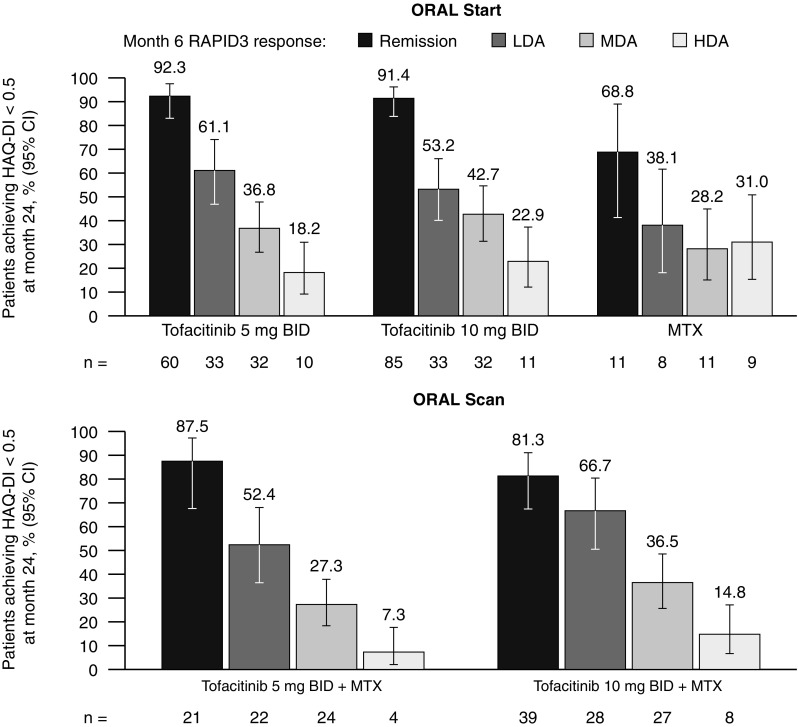
Patients reporting normative HAQ-DI scores (< 0.5) at month 24 stratified by RAPID3 responses at month 6. Disease activity levels for RAPID3 score were defined as remission (≤ 3), LDA (> 3 to ≤ 6; excluding remission), MDA (> 6 to ≤ 12), and HDA (> 12). *BID* twice daily, *CI* confidence interval, *HAQ-DI* Health Assessment Questionnaire-Disability Index, *HDA* high disease activity, *LDA* low disease activity, *MDA* moderate disease activity, *MTX* methotrexate, *RAPID3* Routine Assessment of Patient Index Data 3

Patients in RAPID3 remission or LDA at month 6 were more likely to report normative HAQ-DI scores (< 0.5) at month 24 in both RCTs when compared with the corresponding treatment groups (Fig. 4). In ORAL Start, a higher proportion of patients receiving either dose of tofacitinib reported normative HAQ-DI scores at month 24 compared with MTX across all RAPID3 categories, with the exception of patients in HDA.

#### Patients reporting normative HAQ-DI scores with no radiographic progression

**Fig. 5:**
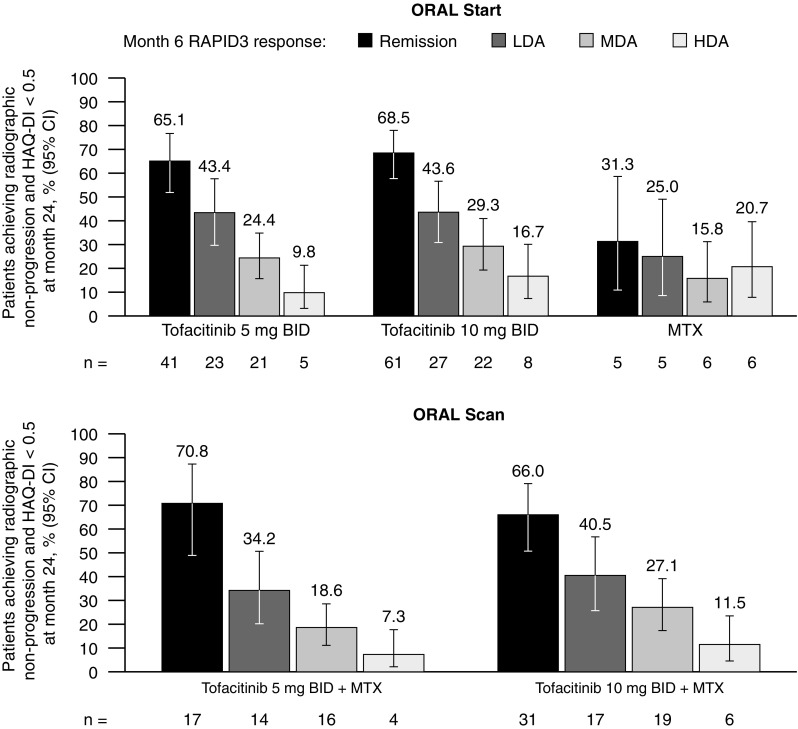
Patients reporting normative HAQ-DI scores and defined with no radiographic progression stratified by RAPID3 responses at month 6. Disease activity levels for RAPID3 score were defined as remission (≤ 3), LDA (> 3 to ≤ 6; excluding remission), MDA (> 6 to ≤ 12), and HDA (> 12). No radiographic progression was defined as a change from baseline in mTSS ≤ 0. *BID* twice daily, *CI* confidence interval, *HAQ-DI* Health Assessment Questionnaire-Disability Index, *HDA* high disease activity, *LDA* low disease activity, *MDA* moderate disease activity, *mTSS* modified Total Sharp Score, *MTX* methotrexate, *RAPID3* Routine Assessment of Patient Index Data 3

In both RCTs, the majority of patients receiving tofacitinib who were in RAPID3 remission at month 6 reported normative HAQ-DI scores and had no radiographic progression at month 24 (Fig. 5). In ORAL Start, a greater proportion of patients receiving either dose of tofacitinib reported normative HAQ-DI scores with no radiographic progression at month 24 than those receiving MTX across all RAPID3 categories, with the exception of patients in HDA.

## Discussion

Established definitions for RA remission by ACR and EULAR depend upon a number of physician-assessed measures [31]. RAPID3 is an easy-to-use, patient-reported measure that is valuable for clinical use [4].

In this post hoc analysis of data from two phase 3 RCTs of tofacitinib in RA, higher rates of RAPID3 remission and LDA were generally observed with both doses of tofacitinib compared with MTX or placebo controls at month 6, with numerically higher rates with 10 mg BID than 5 mg BID. Rates of RAPID3 remission and LDA at month 6 increased at month 24, with the exception of the tofacitinib 10 mg BID group in ORAL Start. The majority of patients who were in remission or LDA at month 6 sustained or improved their RAPID3 response. MTX-naïve patients in ORAL Start receiving either dose of tofacitinib were more likely to attain RAPID3 remission and LDA than those with MTX. In addition, a higher proportion of MTX-naïve patients in ORAL Start achieved RAPID3 remission or LDA with tofacitinib treatment than MTX-IR patients in ORAL Scan. Although this analysis is the first to evaluate RAPID3 in patients receiving tofacitinib, RAPID3 scores have previously been correlated with Disease Activity Score 28 (DAS28), CDAI, and SDAI results [32, 33].

In this analysis, we assessed both radiographic (mTSS) and functional (HAQ-DI) outcomes in line with considerations used to develop the ACR/EULAR remission criteria [34]. As RAPID3 score is based on a pooled index of PROs, including HAQ-DI, correlations between these two measures are expected. The majority of patients in RAPID3 remission at month 6, receiving either dose of tofacitinib, reported normative HAQ-DI scores and had no radiographic progression at month 24. A higher proportion of patients in RAPID3 remission or LDA reported normative HAQ-DI scores and had no radiographic progression at month 24, compared with those in MDA or HDA at month 6. As expected, a higher proportion of patients in RAPID3 remission or LDA at month 6 had improvements in CDAI and reported normative HAQ-DI scores at month 24, compared with those in MDA or HDA at month 6.

A recent study of RAPID3 as a predictor of longer-term RA treatment outcomes with tocilizumab reported that RAPID3 responses at month 12 were able to predict functional and radiographic responses at month 24 [35]. This study also showed that predictability of RAPID3 was comparable to that of SDAI or Boolean remission criteria. This is in agreement with findings reported here, suggesting that RAPID3 remission or LDA at month 6 are associated with better longer-term treatment outcomes with tofacitinib at month 24.

Recently, increased attainment of normative HAQ-DI scores at month 24 was reported by patients who achieved CDAI remission or LDA at month 6 in ORAL Start and ORAL Scan [29]. In the analysis presented here, physical function was more aligned with RAPID3 categories than CDAI responses: numerically, more patients reported normative HAQ-DI scores if they were in RAPID3 remission or LDA than those in CDAI remission or LDA, as previously reported in [27]. Approximately 50% of patients in this analysis, who were in RAPID3 remission at month 6, were also in CDAI remission at month 24. It is also worth noting that a large proportion of patients in RAPID3 MDA (49–68%) at month 6, and up to half of those with HDA (26–52%), still achieved CDAI remission or LDA by month 24 across all treatments. However, complete concordance between CDAI and RAPID3 cannot be expected, given that CDAI considers joint counts and only one PRO, while RAPID3 includes three PROs.

This analysis has a number of limitations. It was conducted post hoc and data were not subjected to multiple comparison adjustments; results should therefore be interpreted carefully alongside other measures of disease activity. The cohort used for this analysis may also not be fully representative of patients in clinical practice. In addition, patients receiving placebo advanced to tofacitinib treatment at months 3 or 6; thus, there was no placebo comparison at month 24. Finally, due to the small numbers within selected patient groups, a large amount of variability was present in some comparisons, resulting in wide and overlapping 95% CIs.

In conclusion, these data demonstrate that patients receiving tofacitinib 5 or 10 mg BID who achieved RAPID3 remission and LDA at month 6 had improved long-term outcomes at month 24, according to CDAI responses and HAQ-DI scores (including the proportion of patients reporting both normative HAQ-DI scores with no radiographic progression), than those in RAPID3 MDA or HDA. Data from this analysis supports the use of RAPID3 as a means to monitor disease activity and longer-term outcomes at the discretion of the physician.
